# Scientific American

**Published:** 1873-08

**Authors:** 


*Scientific American.*

There is no journal coming to our sanctum, the weekly visits of which we more highly value, than the above. It is a journal of science and art. It is, or should be, the inventor's companion, and a journal all will read with interest who believe the world moves, and desire to know how it *does* move. Munn & Co., 37 Park Row, New York. Price, three dollars.

